# Chromosomal complements of some Atlantic Blennioidei and Gobioidei species (Perciformes)

**DOI:** 10.3897/CompCytogen5i4.1834

**Published:** 2011-11-09

**Authors:** Tatiana Barbosa Galvão, Luiz Antonio Carlos Bertollo, Wagner Franco Molina

**Affiliations:** 1 Department of Cell Biology and Genetics, Centro de Biociências, Universidade Federal do io Grande do Norte, Campus Universitário, 59078 – 970, Natal, RN, Brazil; 2 Department of Genetics and Evolution, Universidade Federal de São Carlos, Via Washington Luiz, Km 235, 13565 – 905, São Carlos, São Paulo, Brazil

**Keywords:** Chromosomal evolution, marine fish, Bleniidae, Gobiidae, Labrisomidae

## Abstract

A remarkable degree of chromosomal conservatism (2n=48, FN=48) has been identified in several families of Perciformes. However, some families exhibit greater karyotypic diversity, although there is still scant information on the Atlantic species. In addition to a review of karyotypic data available for representatives of the suborders Blennioidei and Gobioidei, we have performed chromosomal analyses on Atlantic species of the families Blenniidae, *Ophioblennius trinitatis* Miranda-Ribeiro, 1919 (2n=46; FN=64) and *Scartella cristata* (Linnaeus, 1758)(2n=48; FN=50), Labrisomidae, *Labrisomus nuchipinnis* (Quoy & Gaimard, 1824)(2n=48; FN=50) and Gobiidae, *Bathygobius soporator* (Valenciennes, 1837)(2n=48; FN=56). Besides variations in chromosome number and karyotype formulas, Ag-NOR sites, albeit unique, were located in different positions and/or chromosome pairs for the species analyzed. On the other hand, the heterochromatic pattern was more conservative, distributed predominantly in the centromeric/pericentromeric regions of the four species. Data already available for Gobiidae, Blenniidae and Labrisomidae show greater intra- and interspecific karyotypic diversification when compared to other groups of Perciformes, where higher uniformity is found for various chromosome characteristics. Evolutionary dynamism displayed by these two families is likely associated with population fractionation resulting from unique biological characteristics, such as lower mobility and/or specific environmental requirements.

## Introduction

Although karyotypic characteristics for some families of marine fish are already known, information on groups of Perciformes is still significantly disproportionate. Among these, suborders Blennioidei and Gobioidei stand out because of the large number of species they represent.

Suborders Gobioidei, with 2,121 species, and Blennioidei with 732 species, are spread throughout the tropical zone, typically represented by small specimens with low mobility and the ability to withstand changes in temperature and salinity (Nelson 2006).

Species of Blennioidei and Gobioidei investigated (e.g. Cataudela et al.1973; Garcia et al.1987; Ene 2003) have shown sufficient chromosomal peculiarities for species discrimination and understanding of their evolutionary aspects. In some families, such as Blenniidae, Labrisomidae and Gobiidae, sharing cryptic morphological characteristics combined with poor knowledge of the biological characteristics for many species, contributes to the relative taxonomic inaccuracy of this group. As such, cytotaxonomic markers (Garcia et al. 1987; Caputo 1998; Caputo et al. 2001) and phylogenetic analyses based on molecular data (Wang et al. 2001; Thacker 2003; Gysels et al. 2004; Almada et al. 2005) have been increasingly used when assessing their kinship relations. Indeed, it has been suggested that phylogenetic analyses combine molecular and morphological data (Thacker 2003), as well as cytogenetic information. However, in light of the diversity in these groups, solid chromosome data are not yet sufficiently available, with only 7.5% of Bleniidae species and 4.5% of Gobioidei was karyotyped (Table 1). Despite the scarcity of data, a high degree of chromosomal polymorphism has been characterized among Gobiidae, primarily Robertsonian rearrangements (Caputo et al. 1999, Ene 2003), along with others such as tandemfusions and pericentric inversions (Giles et al. 1985; Thode et al. 1985; Amores et al. 1990).

**Table 1.** Cytogenetic data for Blennioidei and Gobioidei (Perciformes).

Suborder/Family	Species	2n	Karyotype formula	FN	References
Blennioidei					
Blenniidae	*Aidablennius sphynx*	48	4m+4sm+40a	56	Cano et al. (1982)
	*Aidablennius sphynx*	48	2st+46a	50	Cataudella and Civitelli (1975)
	*Atrosalarias fuscus*	48	48a	48	Arai and Shiotsuki (1973)
	*Blennius ocellaris*	48	2m+2st+44a	52	Vitturi et al. (1986)
	*Blennius ponticus*	48	16sm+10st+22a	74	Garcia et al. (1987)
	*Blennius yatabei*	48	6sm+12st+30a	66	Arai and Shiotsuki (1974)
	*Coryphoblennius galerita*	48	2m+12sm+34a	62	Garcia et al. (1973)
	*Dasson trossulus*	40	8m+32st/a	48	Arai and Shiotsuki (1974)
	*Istiblennius enoshimae*	48	2m+46a	50	Arai and Shiotsuki (1973)
	*Istiblennius lineatus*	48	48st/a	48	Arai and Shiotsuki (1974)
	*Lipophrys canevai*	48	8st+40a	56	Cataudella and Civitelli (1975)
	*Lipophrys pholis*	46	8m+8sm+30a	62	Garcia et al. (1987)
	*Lipophrys trigloides*	46	4m+4sm+10st+28a	64	Cano et al. (1982)
	*Lipophrys trigloides*	48	2m+6sm+18st+22a	74	Cataudella and Civitelli (1975)
	*Lipophrys trigloides*	48	2m+22sm+2st+22a	74	Garcia et al. (1987)
	*Lipophrys trigloides*	48	2m+6sm+18st+22a	74	Vitturi et al. (1986)
	*Omobranchus elegans*	42	10m+2sm+6st+24a	60	Arai and Shiotsuki (1974)
	*Omobranchus punctatus*	44	4m+40a	48	Arai (1984)
	*Ophioblennius trinitatis*	46	6m+12st+28a	64	Present study
	*Parablennius incognitus* (= *Blennius incognitus*)	48	4st+44a	52	Cano et al. (1982)
	*Parablennius pilicornis* (= *Blennius pilicornis)*	48	8st+40a	56	Catalano et al. (1985)
	*Parablennius gattorugine*	48	2m+4sm+42a	54	Vitturi et al. (1986)
	*Parablennius pilicornis*	48	48a	48	Brum et al. (1992)
	*Parablennius sanguinolentus*	48	12st+36a	60	Cataudella et al. (1973)
	*Parablennius sanguinolentus*	48	20sm+10st+18a	78	Garcia et al. (1987)
	*Parablennius tentacularis*	48	48st/a	48	Vasil’ev (1985)
	*Parablennius tentacularis*	48	1st+47a	49	Carbone et al. (1987)
	*Parablennius tentacularis*	47	1sm+46a	48	Carbone et al. (1987)
	*Salaria fluviatilis*	48	48st/a	48	Cataudella and Civitelli (1975)
	*Salaria pavo*	48	8st+40a	56	Cataudella et al. (1973)
	*Salaria pavo*	48	16sm+14st+18a	78	Garcia et al. (1987)
	*Salaria pavo*	48	2st+46a	50	Vasil’ev (1980)
	*Salarias faciatus*	48	48a	48	Arai and Shiotsuki (1973)
	*Salarias luctuosus*	48	48st/a	48	Arai and Shiotsuki (1974)
	*Scartella cristata* (= *Blennius cristatus)*	48	2st+46a	50	Vitturi et al. (1986)
	*Scartella cristata*	48	2sm+46st/a	50	Brum et al. (1995)
	*Scartella cristata*	48	4st+44a	52	Present study
Gobioidei					
Clinidae	*Clinithracus argentatus*	48	2st+46a	50	Vitturi et al. (1986)
Labrisomidae	*Labrisomus nuchipinnis*	48	2sm+46a	50	Affonso (2000)
	*Labrisomus nuchipinnis*	48	2st+46a	50	Present study
Eleotridae	*Dormitator latifrons*	46	44m/sm+2st/a	90	Uribe-Alcocer et al. (1983)
	*Dormitator maculatus*	46	34m/sm+12st/a	80	Maldonado-Monroy et al. (1985)
	*Dormitator maculatus*	46	40m/sm+6st/a	86	Molina (2005)
	*Dormitator maculatus*	46	14m+28sm+2st+2a(♀) 13m+28sm+3st+2a(♂)	90	Oliveira and Almeida-Toledo (2006)
	*Eleotrioides strigatus*	44	2m+42st/a	46	Arai and Sawada (1974)
	*Eleotris acanthopomus*	46	46st/a	46	Arai and Sawada (1974)
	*Eleotris picta*	52	52a	52	Uribe-Alcocer and Diaz-James (1996)
	*Eleotris pisonis*	46	2m/sm+42st/a	46	Uribe-Alcocer and Diaz-James (1996)
	*Eleotris pisonis*	46	46a	46	Rocon-Stange (1992)
	*Eleotris pisonis*	46	46a	46	Molina (2005)
	*Eleotris muralis*	46	46a	46	Khuda-Bukhsh and Nayak (1990)
	*Mogurnda mogurnda*	46	6sm+40st/a	52	Arai et al. (1974)
	*Mogurnda obscura*	62	-	-	Nogusa (1960)
	*Ophiocara porocephala*	48	48a	48	Arai and Fujiki (1979)
	*Oxyeleotris marmorata*	46	2m+2sm+42a	50	Arai and Fujiki (1979)
Gobiidae	*Aboma latipes*	40	40a	40	Arai and Sawada (1974)
	*Acanthogobius flavimanus*	44	44st/a	44	Arai and Sawada (1974)
	*Acanthogobius flavimanus*	44	36st+8a	80	Arai and Kobayashi (1973)
	*Acanthogobius flavimanus*	44	10m/sm/st+34a	54	Arai and Sawada (1975)
	*Acentrogobius pflaumi*	50	48m/sm+2st/a	98	Nogusa (1960)
	*Amblygobius albimaculatus*	44	2m+42st/a	46	Nishikawa et al. (1974)
	*Aphia minuta*	44	44a	44	Caputo et al. (1999)
	*Aphia minuta*	43	42a+1st	42	Caputo et al. (1999)
	*Aphia minuta*	42	1m+1st+40a	44	Caputo et al. (1999)
	*Aphia minuta*	42	1M+1m+40a	44	Caputo et al. (1999)
	*Aphia minuta*	41	2M+1st+38a	44	Caputo et al. (1999)
	*Apocryptes bato*	46	24m+10sm+12a	80	Nayak and Khuda-Bukhsh (1987)
	*Apocryptes lanceolatus*	38	14m+22sm+2st	76	Nayak and Khuda-Bukhsh (1987)
	*Awaous grammepomus*	46	46st/a	46	Khuda-Bukhsh and Barat (1987)
	*Awaous tajasica*	46	46a	46	Stange and Passamani (1986)
	*Bathygobius fuscus*	48	48a	48	Arai and Sawada (1975)
	*Bathygobius soporator*	48	2m+46a	50	Brum et al. (1996)
	*Bathygobius soporator*	48	2m/sm+46a	50	Cipriano et al. (2002)
	*Bathygobius soporator*	48	2m+6st+40a	56	Present study
	*Bathygobius stellatus*	46	2st+44a	48	Vasil’ev (1985)
	*Bathygobius stellatus*	47	1sm+2st+43a	49	Vasil’ev (1985)
	*Boleophthalmus boddaerty*	46	46m/sm	92	Subrahmanyan (1969)
	*Boleophthalmus glaucus*	46	12m+20sm+2st+12a	80	Manna and Prasad (1974)
	*Boleophthalmus pectinirostrus*	46	46st/a	46	Arai and Sawada (1975)
	*Bostrichthys sinensis*	48	4m/sm+44a	52	Arai et al. (1974)
	*Chaenogobius annularis*	44	18sm+26st/a	62	Arai and Sawada (1975)
	*Chaenogobius annularis*	44	36m/sm+8a	80	Arai et al. (1974)
	*Chaenogobius annularis*	44	44a	44	Nogusa (1960)
	*Chaenogobius castaneus*	44	36m/sm/st+8a	80	Nishikawa et al. (1974)
	*Chaenogobius isaza*	44	12sm+32st/a	56	Arai and Sawada (1975)
	*Chaenogobius urotaenia*	44	-	-	Nogusa (1960)
	*Chaenogobius urotaenia*	42	14sm+28a	56	Yamada (1967)
	*Chasmichthys dolichognatus*	44	44st/a	44	Arai and Sawada (1975)
	*Chaenogobius gulosus*	44	44st/a	44	Arai and Sawada (1975)
	*Chaenogobius gulosus*	44	16m/sm/st+28a	60	Nishikawa et al. (1974)
	*Ctenogobius criniger*	50	34m/sm+6st+10a	90	Arai and Sawada (1974)
	*Gillichthys mirabilis*	44	12sm+32a	56	Chen and Ebeling (1971)
	*Gillichthys seta*	44	6m+14sm+24a	64	Chen and Ebeling (1971)
	*Glossogobius fasciatopunctatus*	44	10m+28sm+2st+4a	84	Fei and Tao (1987)
	*Glossogobius giuris*	46	46a	46	Rishi and Singh (1982)
	*Gobiodon citrinus*	44	2m+42st/a	46	Arai and Sawada (1974)
	*Gobiodon citrinus*	43	1m+42st/a	44	Arai and Sawada (1974)
	*Gobiodon quinquestrigatus*	44	44a	44	Arai and Fujiki (1979)
	*Gobiodon rivulatus*	44	44a	44	Arai and Fujiki (1979)
	*Gobioides rubicundus*	46	2m+26sm+10st+8a	84	Manna and Prasad (1974)
	*Gobionellus shufeldti*	48	48a (♀)	48	Pezold (1984)
	*Gobionellus shufeldti*	47	46a+1m (♂)	48	Pezold (1984)
	*Gobiosoma macrodon*	38	38a	38	Musammil (1974)
	*Gobiosoma zebrella*	38	38a	38	Musammil (1974)
	*Gobius abei*	46	-	-	Nogusa (1960)
	*Gobius bucchichi*	44	2sm+42a	46	Thode and Alvarez (1983)
	*Gobius cobitis*	46	46a	46	Caputo et al. (1997)
	*Gobius cruentatus*	46	2st+44a	48	Thode and Alvarez (1983)
	*Gobius fallax*	38	8m/sm+30a	46	Thode et al. (1988)
	*Gobius fallax*	39	7m/sm+32a	46	Thode et al. (1988)
	*Gobius fallax*	40	6m/sm+34a	46	Thode et al. (1988)
	*Gobius fallax*	40	7m/sm+33a	47	Thode et al. (1988)
	*Gobius fallax*	41	5m/sm+36a	46	Thode et al. (1988)
	*Gobius fallax*	42	4m/sm+38a	46	Thode et al. (1988)
	*Gobius fallax*	43	3m/sm+40a	46	Thode et al. (1988)
	*Gobius niger*	52	2m+4sm+16st+30a	74	Vitturi and Catalano (1989)
	*Gobius niger*	51	3m+4sm+16st+28a	74	Caputo et al. (1997)
	*Gobius niger*	50	4m+4sm+16st+26a	74	Caputo et al. (1997)
	*Gobius niger*	49	5m+4sm+16st+24a	74	Caputo et al. (1997)
	*Gobius paganellus*	48	2sm+46a	50	Caputo et al. (1997)
	*Gobius similis*	44	?		Nogusa (1960)
	*Gobiusculus flavescens*	46	6m/sm+40a	52	Klinkhardt (1992)
	*Luciogobius grandis*	44	?		Arai (1981)
	*Luciogobius guttatus*	44	?		Arai and Kobayashi (1973)
	*Mesogobius batrachocephalus*	30	16m+14a	46	Ivanov (1975)
	*Neogobius cephalarges*	46	46a	46	Vasil’ev (1985)
	*Neogobius constructor*	42	4m/sm+38a	46	Vasil’ev and Vasil'yeva (1994)
	*Neogobius cyrius*	36	structural polymorphism		Vasil’ev and Vasil'yeva (1994)
	*Neogobius fluviatilis*	46	46a	46	Vasil’ev (1985)
	*Neogobius eurycephalus*	32	12m+2sm+18a	46	Ene (2003)
	*Neogobius eurycephalus*	31	13m+2sm+16a	46	Ene (2003)
	*Neogobius eurycephalus*	30	14m+2sm+14a	46	Ene (2003)
	*Neogobius gymnotrachelus*	46	46a	46	Vasil’ev and Grigoryan (1992)
	*Neogobius kessleri*	46	46a	46	Vasil’ev (1985)
	*Neogobius melanostomus*	46	46a	46	Vasil’ev (1985)
	*Neogobius rhodionovi*	46	46a	46	Vasil’ev and Vasil'yeva (1994)
	*Odontamblyops rubicundus*	46	4m+16sm+26st/a	66	Arai and Sawada (1975)
	*Padogobius martensi*	46	1m+3sm+2st+40a	52	Cataudella et al. (1973)
	*Parioglossus raoi*	46	46st/a	46	Webb (1986)
	*Periophthalmus cantonensis*	46	18m+12sm+16st/a	76	Arai and Sawada (1975)
	*Pomatoschistus lozanoi*	37	3m+12sm+10st+12a	62	Webb (1980)
	*Pomatoschistus microps*	46	4m+16sm+20st+6a	86	Klinkhardt (1989)
	*Pomatoschistus minutus*	46	4m+16sm+16st+10a	82	Klinkhardt (1989)
	*Pomatoschistus minutus*	46	18sm+18st+10a	82	Klinkhardt (1992)
	*Pomatoschistus norvegicus*	32	10m+10sm+8st+4a	60	Webb (1980)
	*Pomatoschistus pictus*	46	22m/sm+12st+12a	80	Klinkhardt (1992)
	*Proterorhinus marmoratus*	46	46a	46	Rab (1985)
	*Pterogobius elapoides*	44	14sm+30st	88	Arai and Kobayashi (1973)
	*Pterogobius zonoleucus*	44	14sm+30st	88	Arai and Sawada (1975)
	*Quietula guaymasiae*	42	6m+4sm+32a	52	Cook (1978)
	*Quietula y-cauda*	42	42a	42	Cook (1978)
	*Rhinogobius brunneus*	44	44a	44	Nishikawa et al. (1974)
	*Rhinogobius flumineus*	44	44a	44	Arai and Kobayashi (1973)
	*Rhinogobius giurinus*	44	44a	44	Nishikawa et al. (1974)
	*Rhodoniichthys laevis*	42	16m/sm+26st	84	Arai et al. (1974)
	*Sicyopterus japonicus*	44	10m+10sm+24a	64	Arai and Fujiki (1979)
	*Synechogobius hasta*	44	2m+42st/a	46	Arai and Sawada (1975)
	*Tridentiger obscurus*	44	10m/sm+34a	54	Arai et al. (1974)
	*Tridentiger trigonocephalus*	44	28m/sm/st+16a	72	Arai et al. (1973)
	*Tridentiger trigonocephalus*	46	16sm+6st+24a	68	Fei and Tao (1987)
	*Trypauchen vagina*	46	12m+6sm+10st+18a	74	Khuda-Bukhsh (1978)
	*Tukugobius flumineus*	44	44a	44	Nadamitsu (1974)
	*Zosterisessor ophiocephalus* (= *Gobius ophiocephalus)*	46	46a	46	Vasil’ev (1985)
	*Zosterisessor ophiocephalus* (= *Gobius ophiocephalus)*	45	1st+45a	47	Vasil’ev (1985)
	*Zosterisessor ophiocephalus*	46	2m/sm+44a	48	Caputo et al. (1996)

The present study focuses on the karyotypic characterization of some Atlantic species of the families Blenniidae, *Ophioblennius trinitatis* Miranda-Ribeiro, 1919 and *Scartella cristata* (Linnaeus, 1758), Labrisomidae, *Labrisomus nuchipinnis* (Quoy & Gaimard, 1824)and Gobiidae, *Bathygobius soporator* (Valenciennes, 1837), through conventional chromosomal analysis, characterization of nucleolar organizer regions (Ag-NORs) and the distribution pattern of C-positive heterochromatin (C-banding) in chromosomes, discussing evolutionary aspects.

## Material and methods

A total of 25 specimens of *Ophioblennius trinitatis* (7♂, 4♀ and 14 indeterminate), 11 specimens of *Scartella cristata* (4♂, 5♀ and 2 indeterminate), 13 specimens of *Labrisomus nuchipinnis* (4♂, 4♀ and 5 indeterminate) and 12 specimens of *Bathygobius soporator*, (5♂, 5♀ and 2 indeterminate) were used for chromosome analysis. *Ophioblennius trinitatis* specimens came from the coast of Rio Grande do Norte (5°13'1.73"S; 35°9'57.85"W), northeastern Brazil (n=1), and the Saint Peter and Saint Paul (n=8) (00°55'02"N; 29°20'42"W) and Fernando de Noronha (n=16) (3°52'11"S; 32°26'13"W) archipelagos. The remaining specimens were collected on the coast of Rio Grande do Norte. Individuals were previously submitted to mitotic stimulation with compound attenuated antigens, for 24 to 48 hours (Molina 2001, Molina et al. 2010), anesthetized with clove oil (Eugenol) and sacrificed for the removal of anterior kidney fragments. Sexing of specimens was performed by macroscopic and microscopic examination of the gonads. Chromosome preparations were obtained from kidney cells (Gold et al. 1990). Nucleolar organizer regions (NORs) were identified by stain with silver nitrate - Ag-NORs (Howell and Black 1980) and C-positive heterochromatin sites through C-banding (Sumner 1972).

Metaphase preparations were examined and photographed on an Olympus BX50 photomicroscope, using an Olympus DP70 digital camera system. Chromosomes were classified according to the position of the centromere in metacentrics (m), submetacentrics (sm), subtelocentrics (st) and acrocentrics (a) (Levan et al. 1964) and organized in order of decreasing size. The chromosome formula and FN (fundamental number or number of chromosomal arms) were established for each species, considering acrocentric chromosomes with a single arm and the remaining chromosomes exhibiting two arms.

## Results

### Cytogenetic analyses of Blenniidae species (Blennioidei)

**Figure 1.**
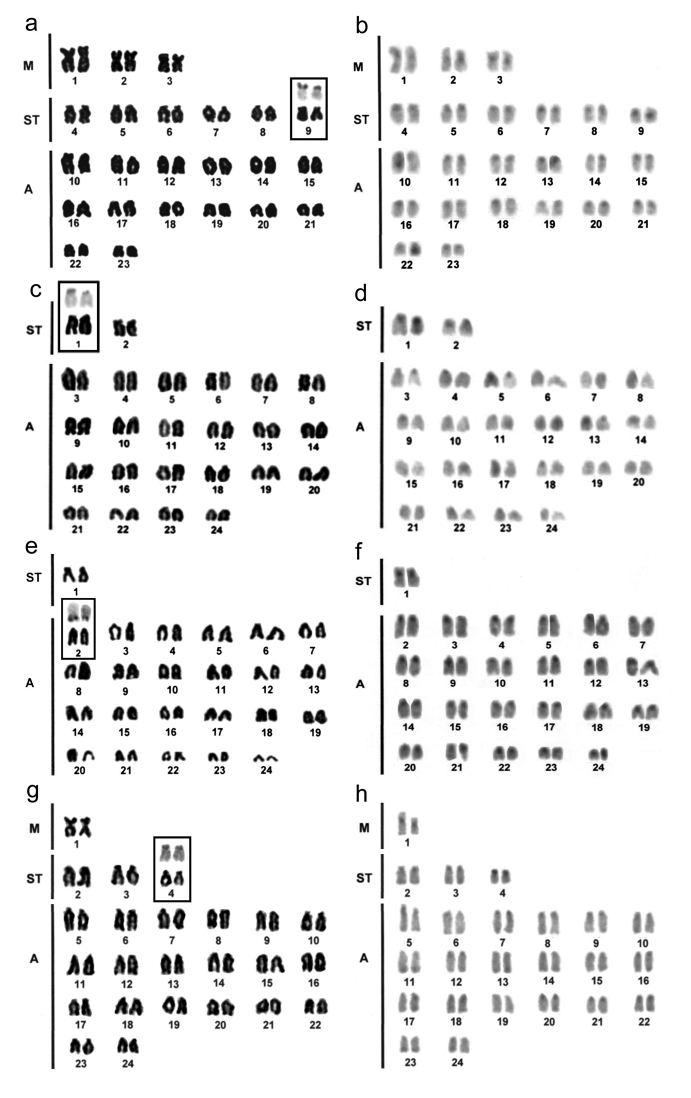
Karyotypes underGiemsa staining **a, c, e, g** and C-banding **b, d, f, h** of *Ophioblenius trinitatis*; **a, b** *Scartella cristata*; **c, d** *Labrisomus nuchipinnis*; **e, f** and *Bathygobius soporator*; **g, h** Ag-NOR-bearing chromosome pairs are highlighted.

*Ophioblennius trinitatis*showed 2n=46, with a chromosome formula equal to 6m+12st+28a (FN=64), irrespective of sex. Although chromosomes showed a gradual decline in size, the smallest acrocentric pairs corresponded to approximately one-third of the largest metacentric pairs. Nucleolar organizer regions are located in the terminal portions of the short arm on pair 9, the smallest subtelocentric pair. C-positive heterochromatin is discretely located in the centromeric/pericentromeric region of the chromosomes (Fig. 1a, b).

*Scartella cristata* showed 2n=48 chromosomes, with a chromosome formula equal to 4st+44a (FN=52). The karyotype also displays a gradual reduction in chromosome size. However, the largest chromosome pair exhibits only double the size in relation to the smallest karyotype pair. Ribosomal sites are located on the terminal portions of the short arms on chromosome pair 1. C-positive heterochromatin is also reduced and located in the centromeric regions of chromosomes (Fig. 1c, d).

### Cytogenetic analyses of Labrisomidae and Gobiidae species (Gobioidei)

*Labrisomus nuchipinnis* (Labrisomidae) showed 2n=48 chromosomes with a chromosome formula of 2st+46a (FN=50), showing relatively more differentiated size between the largest and smallest chromosomes of the karyotype. Nucleolar organizer regions are in the terminal portions of the long arms on pair 2, corresponding to the largest pair of acrocentric chromosomes. C-positive heterochromatin was showed in the centromeric/pericentromeric region of all chromosome pairs, in relatively conspicuous blocks (Fig. 1e, f).

*Bathygobius soporator* (Gobiidae) also displayed the karyotype composed of 2n=48 chromosomes, but with the chromosome formula distinct from that of *Labrisomus nuchipinnis*, specifically, 2m+6st+40a (FN=56). Size difference between the largest and smallest chromosomes of the karyotype was far less pronounced. Ribosomal sites were on the terminal portions of the short arms on chromosome pair 4. C-banding showed discrete heterochromatic regions in the centromeric regions of most chromosomes and telomeric regions of some acrocentric pairs (Fig. 1g, h).

## Discussion

Though many perciform families display a conserved karyotype pattern, with 2n=48 acrocentric chromosomes, some groups demonstrate dynamic tendencies in relation to chromosome evolution (Molina 2007). Much of identifiable chromosome diversity is attributed to pericentric inversions, the most common mechanism of chromosome evolution in this order (Galetti et al. 2000, 2006).

Representatives of the suborder Blennioidei (e.g., Carbone et al. 1987) and Gobioidei (e.g., Arai and Sawada 1974, 1975; Thodeet al. 1988; Oliveira and Almeida-Toledo 2006) stand out for their greater karyotype variability and diversity. This includes species with conserved karyotyes and those that are highly diversified.

Within the Blennioidei, the Blenniidae, a monophyletic family, is divided into six tribes including Salariini and Parablenniini which, in turn, include the Atlantic species *Ophioblennius trinitatis* and *Scartella cristata* respectively (Nelson 2006). Comparisons of mitochondrial DNA sequences in samples of *Ophioblennius* Gill, 1860 collected throughout the Atlantic suggest that the genus consists of six distinct lineages. One of these corresponds to species found in the Pacific, while the rest are recorded in the biogeographic provinces of the Atlantic: Brazilian, Caribbean, Mid-Atlantic, Sao Tome and Azores/Cape Verde (Muss et al. 2001). Chromosome characteristics reported here for *Ophioblennius trinitatis* are the first for the genus, exhibiting 2n=46, 6m+12st+28a and FN=64. The relatively low diploid number and higher fundamental number in relation to the mean of other species of Blenniidae (Table 1), as well as the presence of large metacentric chromosomes, suggests pericentric inversion events and the occurrence of Robertsonian translocation involving two of its chromosome pairs. In turn, *Scartella cristata*,while also belonging to the family Blenniidae, has a distinct karyotype of 2n=48, 4st+44a and FN=52. Thus, *Scartella cristata* differs from *Ophioblennius trinitatis* in that it contains an extra pair of chromosomes, lacks metacentric chromosomes and has different numbers of subtelocentric and acrocentric chromosomes in the karyotype. The karyotype of the *Scartella cristata* population studied here differs from the karyotypes previously described for the coastal population of Rio de Janeiro (SE Brazil), with 2sm+46st/a (Brum et al. 1994), and the Mediterranean population, with 2st+46a (Vitturi et al. 1986). Nevertheless, despite the growing number of discordant karyotype descriptions between populations on the NE and SE coasts of Brazil, one cannot rule out that these differences may arise from the difficulty in precisely defining types of cryptic chromosomes in the karyotype of this species.

In spite of displaying relative diversity in chromosome structure, only 18.5% of Blennioidei species exhibit differences in the basal diploid number, 2n=48 chromosomes. As shown in table 1, diploid numbers for representatives of this suborder vary between 2n=40, found in *Dasson trossulus* (Jordan & Snyder, 1902)(Arai and Shiotsuki 1974) and 2n=52 in *Gobius niger* Linnaeus, 1758 (Vitturi and Catalano 1989), but with a conspicuous modal value of 2n=48.

In contrast to Blennioidei, suborder Gobioidei shows much more dynamic karyotype evolution, demonstrating highly variable karyotype patterns, where the diploid number ranges from 2n=30 for *Neogobius eurycephalus* (Kessler, 1874) (Ene 2003), to 2n=62 in *Mogurnda mogurnda* (Richardson, 1844) (Nogusa 1960). Cytogenetic data for 95 species show that only 9.6% have 2n=48 chromosomes, whereas the highest frequencies observed correspond to 2n=46 in 40% of species investigated, and 2n=44 in 32% (Table 1). As such, both Gobioidei species studied here are included in the group showing 2n=48 chromosomes, *Labrisomus nuchipinnis* with 2st+46a and FN=50 and *Bathygobius soporator* with 2m+6st+40a and FN=56. Thus, *Bathygobius soporator* differs from *Labrisomus nuchipinnis* in the presence of metacentric chromosomes and different numbers of subtelocentric and acrocentric chromosomes in the karyotype.

Among chromosome rearrangements involved in karyotypic differentiation of Gobiidae, Robertsonian fusions stand out, and are likely the most common event in this group (Amores et al. 1990; Galetti et al. 2000). However, other more complex changes in karyotypic structure (Thode et al. 1988; Vitturi and Catalano 1989; Caputo et al. 1997; Caputo et al. 1999), as well as the presence of different sex chromosomes (e.g., Pezold 1984; Baroiller et al. 1999), can also be observed, corroborating the high dynamic evolution that characterizes suborder Gobioidei. It has been suggested that the baseline/ancestral karyotype for Gobiidae would consist of 2n=46 acrocentric chromosomes (Vasil’ev and Grigoryan 1993), from which an increase in bi-brachial chromosomes would characterize more derived karyotypes. Based on this proposal, *Bathygobius soporator* (FN=56) would experience a greater number of structural rearrangements during its karyotypic evolution process in relation to *Labrisomus nuchipinnis* (FN=50).

**Figure 2.**
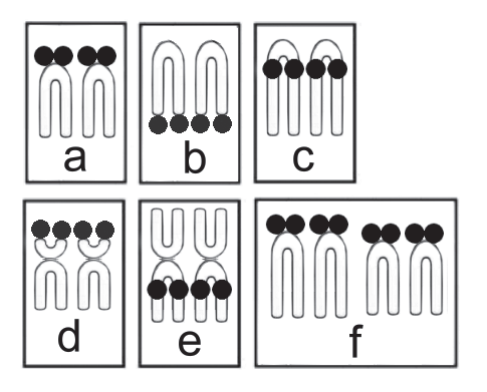
Ag-NOR phenotypes **a–f** described in species of Gobiidae. Ag-NORs sites described in the karyotypes of Gobiidae species were found **a** in the telomeric region on the short arm of a single pair of acrocentric chromosomes **b** in the telomeric region on the long arm of a single pair of acrocentrics **c** in the interstitial/pericentromeric region on the long arm of a single pair of acrocentric chromosomes **d** in the telomeric region on the short arm of a single subtelocentric pair **e** in the interstitial/pericentromeric region on the long arm of a single metacentric pair and **f** in the telomeric regions on the short arms of two acrocentric chromosome pairs.

Location and frequency of Ag-NOR sites are efficient cytotaxonomic markers in many groups of fish (Caputo 1998). Among species of Gobiidae, at least six different arrangement patterns for nucleolar organizer regions have been identified (Fig. 2), which supports the occurrence of intense karyotypic diversification mechanisms in this group. Thus, Ag-NOR sites can be found (a) in the telomeric region on the short arm of a single pair of acrocentric chromosomes, as in *Gobius fallax* Sarato, 1889 (Thode et al. 1983) and *Gobius paganellus* Linnaeus, 1758 (Caputo 1998); (b) in the telomeric region on the long arm of a single pair of acrocentrics, such as in *Zosterisessor ophiocephalus* (Pallas, 1814) (Caputo 1998); (c) in the interstitial/pericentromeric region on the long arm of a single pair of acrocentric chromosomes, as seen in *Proterorhinus marmoratus* (Pallas, 1814) (Ráb 1985) and *Gobius cobitis* Pallas, 1814 (Caputo 1998); (d) in the telomeric region on the short arm of a single subtelocentric pair, described in *Bathygobius soporator*;(e) in the interstitial/pericentromeric region on the long arm of a single metacentric pair, observed in *Neogobius eurycephalus* (Ene 2003); and (f) in the telomeric regions on the short arms of two acrocentric chromosome pairs, recorded in *Gobiusculus flavescens* (Fabricius, 1779) (Klinkhardt 1992).

Few data are available on ribosomal sites for Labrisomidae. Ag-NORs in *Labrisomus nuchipinnis* exhibit the phenotype (b) described above, in addition to both species of Blenniidae, *Ophioblennius trinitatis* and *Scartella cristata*, which may suggest an ancestral condition for this location.

In contrast, other chromosome characteristics, such as C-positive heterochromatin distribution, may be more conserved. This occurs in several species of Percifomes where discrete blocks are preferentially located in the centromeric/pericentromeric regions of chromosomes (Molina 2007). This pattern is repeated in *Scartella cristata*, *Ophioblennius trinitatis* and *Labrisomus nuchipinnis*, as well as in some Gobiidae, such as *Gobius cobitis*, *Zosterisessor ophiocephalus* and *Neogobius eurycephalus* (e.g. Caputo et al. 1997; Ene 2003). In *Bathygobius soporator*,in addition to centromeric/pericentromeric regions, heterochromatic sites are also observed in terminal regions of some chromosomes. This arrangement has already been described for other Gobiidae, including *Gobius paganellus* and *Gobius niger*, where pericentromeric and telomeric heterochromatic regions are distributed among almost all chromosomes (Amores et al. 1990; Caputo et al.1997).

Moreover, karyotypic diversity present in Gobioidei is increased by the occurrence of chromosome polymorphisms frequently observed in this group. This is particularly evident in several examples of intraspecific karyotypic variability, as well as polymorphisms involving different types of chromosome rearrangements, such as in *Gobius niger* (Vitturi and Catalano 1989; Caputo et al. 1997) and *Gobius fallax* (Thode et al. 1988). Data obtained for the paedomorphic Gobiidae *Aphia minuta* (Risso, 1810) also show variations in the diploid number and chromosome formula, resulting in five different cytotypes (2n=41–44 and FN=42-44) (Caputo et al. 1999). Similar karyotypic variability was reported in *Neogobius eurycephalus*, where three specific cytotypes (2n=30, 31 and 32) were associated to the occurrence of centric fusions (Ene 2003). All these examples demonstrate clear chromosomal dynamism, with possible transitions to new karyotype patterns.

In fact, karyotypic diversity among Blennioidei and Gobioidei seems to accompany phyletic diversification of these groups. This is a result of vicariant factors (Pampoulie et al. 2004) and could be favored by their low dispersive potential (Fanta 1997), as well as ecological specificities that favor population fractionation in this family (Huyse et al. 2004). The present study also highlight the importance of ribosomal sites as effective chromosomal markers in the further cytogenetic studies in gobiids species.
